# Low-density lipoprotein cholesterol levels and treatment intensity in secondary prevention of patients with ischaemic heart disease in the primary care setting: a real-world data registry study

**DOI:** 10.3399/BJGPO.2024.0220

**Published:** 2025-06-12

**Authors:** Núria Sánchez-Ruano, Anna Fibla-Matamoros, Carles Falces, Encarna Sánchez, Antoni Sisó-Almirall, Luis González-de Paz

**Affiliations:** 1 Consorci d’Atenció Primària de Salut Barcelona Esquerra, Barcelona, Spain; 2 Cardiovascular Institute, Hospital Clínic de Barcelona, Barcelona, Spain; 3 Research Group on Atherosclerosis, Coronary Disease and Heart Failure, Institut d’Investigacions Biomèdiques August Pi i Sunyer (IDIBAPS), Barcelona, Spain; 4 Primary Healthcare Transversal Research Group, IDIBAPS, Barcelona, Spain; 5 Department of Medicine, University of Barcelona, Barcelona, Spain; 6 Department of Public Health, Mental Health and Mother and Child Health, University of Barcelona, Barcelona, Spain

**Keywords:** cholesterol, coronary disease, secondary prevention, lipids

## Abstract

**Background:**

Monitoring low-density lipoprotein cholesterol (LDL-C) and prescribing appropriate treatment is crucial for secondary prevention in primary care.

**Aim:**

To study LDL-C levels and treatments for patients with ischaemic heart disease according to target recommendations and assess factors influencing prescribed drug intensity.

**Design & setting:**

A cross-sectional study was undertaken. We examined electronic health records of patients with ischaemic heart disease from three primary care centres in Spain.

**Method:**

LDL-C levels were assessed using the most recent registry, and LDL-C-lowering treatments were categorised by their theoretical efficacy. Factors associated with LDL-C target attainment were analysed using univariate and multivariate regression models. Prescription intensity was studied with ordinal logistic regression models.

**Results:**

We studied 1936 patients, 14.88% of whom received no LDL-C-lowering treatment. The percentages of patients who achieved LDL-C thresholds of<70 mg/dl and<55 mg/dl were 35.0% and 12.65%, respectively. The factor associated with the <55 mg/dl threshold was type 2 diabetes mellitus (odds ratio [OR] 0.55, 95% confidence interval [CI] = 0.42 to 0.73), with males showing better LDL-C levels (OR 0.34, 95% CI = 0.23 to 0.51). Males had higher-intensity prescriptions (OR 1.57, 95% CI = 1.27 to 1.94) and older patients had lower-intensity treatments (OR 0.96, 95% CI = 0.95 to 0.97).

**Conclusion:**

Increased LDL-C drug treatment improvement, monitoring, and adherence to guideline recommendations are necessary for patients with ischaemic heart disease. Sex and age are potential factors associated with inadequate lipid-lowering treatment intensity and poor LDL-C control that might worsen cardiovascular outcomes in high-risk patients, leading to avoidable inequity among patients who visit the primary health setting.

## How this fits in

Maintaining low-density lipoprotein cholesterol (LDL-C) levels through appropriate treatment is essential for heart attack prevention. Based on clinical guidelines, our study assessed LDL-C management and drug intensity in patients with heart disease. Results revealed significant undertreatment, especially among females, with LDL-C levels falling short of targets for secondary prevention. Age and sex disparities highlight the need to address frailty in older patients with multimorbidity when managing cardiovascular care.

## Introduction

LDL-C is key to secondary prevention in patients with ischaemic heart disease.^1^ Recommendations for lowering LDL-C levels include lifestyle modifications such as smoking cessation, increased physical activity, improved diet,^2^ and treatment with a high-intensity statin.^3^ Lipid-lowering drug treatments in secondary prevention have shown a decrease of 1 mmol/l (40 mg/dl) in LDL-C from the initial LDL-C values irrespective of baseline LDL-C blood levels, leading to a 24% decrease in the incidence of new major adverse cardiovascular events.^4^

Drug treatments targeting LDL-C reduction primarily involve statins, ezetimibe, PCSK9 inhibitors (PCSK9i), and the most recent bempedoic acid and PCSK9-interfering mRNAs (PSCK9m). High-intensity statin therapy is the first choice for LDL-C therapy, allowing a decrease in LDL-C of 50% and, if combined with ezetimibe, safely improving LDL-C reduction by an additional 15%–30%.^5,6^ In patients with intolerance, a moderate-intensity statin plus ezetimibe is recommended. Treatments with PCSK9i (very high or extremely high cholesterol-lowering therapy) are an alternative when the LDL-C reduction target is >60% or >80% of the baseline. However, the prescription of PSCK9i is recommended for the secondary prevention of cardiovascular disease if the LDL-C target is not achieved when using high-potency statins combined with ezetimibe or in statin-intolerant patients.^7^ Furthermore, in some countries, such as Spain, the healthcare system reimburses the PSCK9i or the PSCK9m treatments to patients with LDL-C levels >100 mg/dl despite treatment with statins at the maximum tolerated dose. However, these treatments are exclusively prescribed in hospitals and are unavailable in primary care settings.

In 2019, the European Society of Cardiology (ESC) and the European Atherosclerosis Society (EAS) introduced a new guideline on managing dyslipidemia for the secondary prevention of cardiovascular disease (CVD). The guideline targets LDL-C levels of <55 mg/dl (<1.4 mmol/l) and a reduction of at least 50% in baseline LDL-C values.^8^ Secondary prevention with LDL-C-lowering therapy is also recommended in patients with multimorbidity and older patients unless the patient’s status is end of life or with moderate or severe frailty.^9^ Despite recommendations, recent studies reported a low proportion of patients attaining LDL-C targets in high-risk patients.^10,11^ However, these studies did not evaluate statin prescribing according to patient characteristics and comorbidities. Therefore, in this study, we examined LDL-C levels and LDL-C lipid-lowering treatments in patients with ischaemic heart disease based on the 2016 and 2019 ESC and EAS guideline recommendations.^8,12^ Secondarily, we evaluated the associated factors based on the prescribed drug intensity for the LDL-C.

## Method

### Design and context

In Spain, the health system jurisdiction assigns citizens to a primary healthcare centre.^13^ We conducted a cross-sectional study in three primary healthcare centers in Barcelona (Spain). The study population comprised 97 749 individuals aged ≥15 years assigned to these centres in 2023, of whom 87.69% (*n* = 85 717) had a health record registry.

### Participants and inclusion criteria

In January 2023, we included all patients with a chronic ischaemic heart disease registry, previous acute myocardial infarction, or ischaemic angina (International Classification of Diseases, tenth revision [ICD-10] codes I25.9, I20.0, or I21.9, respectively). These codes were selected based on their common use by clinicians to document chronic ischaemic heart disease.

### Variables

We collected data from electronic health records on hypertension (ICD-10 code: I10), type 2 diabetes mellitus (ICD-10 code: E11.9), dyslipidemia (ICD-10 codes: E78, E78.1–5, and E78.9), chronic kidney disease (ICD-10 codes: N18 to E18.9), chronic obstructive pulmonary disease (COPD) (ICD-10 code: F4), chronic heart failure (ICD-10 codes: I50.0–9), anxiety or depression (ICD-10 codes: F41.9 or F32), tobacco smoking, and the adjusted morbidity groups (AMG) index, which is an indicator based on patient diagnoses, risk of hospitalisations, and prescriptions. The AMG index is calculated from the entire population in Catalonia (>7 million); the 85th percentile corresponds to the high-risk morbidity group, representing 5% of the population requiring high-intensity care;^14^ the functional dependency on activities of daily living as registered by the Barthel Index;^15^ and the total number of actively prescribed drugs as recorded in the electronic health records system.

We extracted the latest biochemical serum values: LDL-C, total cholesterol, high-density lipoprotein cholesterol, triglycerides, and HbA1c. Consultations with GPs and nurses were based on the last year before data extraction. We evaluated ongoing prescribed LDL-C-lowering treatments during data extraction, categorising them by their theoretical efficacy:^16^ low-intensity (LDL-C reduction <30%), mild-intensity (30% to <50%), high-intensity (50% to <60%), and very high-intensity therapy (60% to 80%). Supplementary Table S1 details the drug classification by intensity.

### Source of data

We extracted the data from an electronic health record registry containing diagnoses, health problems, and clinical indicators documented by primary healthcare physicians and nurses during routine clinical follow-up. A structured query languagxe query was used to retrieve data on all variables for the study. The data were extracted in January 2023.

### Statistical analysis

LDL-C-lowering drug treatment was categorised according to the average reduction intensity, including all possible combinations of treatments available in primary health care, ranging from low to high intensity.^16^ In cases with missing LDL-C, it was determined with the Friedewald formula, if applicable.^17^

Patients were described according to active LDL-C drug treatment status. Differences between the groups with active and without LDL-C-lowering therapy were studied with univariate linear or logistic regression models. To study factors associated with LDL-C target attainment according to the 2016 and 2019 ESC and EAS guidelines, first, we fitted univariate linear or logistic regression models with the target attainment as the response variable and the following explanatory variables: age, sex, type 2 diabetes mellitus, tobacco smoke, hypertension, chronic kidney disease, dyslipidemia, and obesity. Subsequently, a multivariate logistic model was fitted adjusting by all factors that showed statistical significance (*P*<0.05) on the univariate linear or logistic regression models.

The LDL-C drug treatment intensity analysis was based on patient characteristics and risk factors, and was analysed with an ordinal logistic regression model, with treatment intensity as a response variable. The variables that showed statistical significance (*P*<0.05) were included in a multivariate ordered logistic model to examine the association adjusted by all factors for the intensity of LDL-C-lowering drug treatment. Finally, the adjusted associations of statistically significant variables were analysed with effect plots. All results are presented as odds ratios (ORs) with their corresponding 95% confidence intervals (CIs) and *P*-values. The analysis was performed with CRAN R software (version 4.4.2).

## Results

We found 1936 patients with a registry of ischaemic heart disease, resulting in a combined prevalence of 2.26% (95% CI = 2.16% to 2.36%) and 14.88% (*n* = 288) not receiving LDL-C-lowering treatment (Table 1). Most patients were male (70.40%, *n* = 1363), with a mean age of 74.98 years (standard deviation [SD] 11.00). The mean age at which the disease was registered was 65.26 years (SD 11.63), the most recent LDL-C measurement was recorded on average 1.27 years before data extraction (SD 1.37; interquartile range 0.00 to 10.53 years; data not shown), and 79.29% of patients were classified as having high comorbidity status (Table 1). Data on dependency in activities of daily living were documented for 1126 (58.16%) patients, with 162 (8.37%) having severe to total dependence. The prevalence of arterial hypertension was 62.65%, that of type 2 diabetes mellitus was 31.30%, that of chronic kidney disease was 29.44%, and that of active smokers was 10.28%. Patients without LDL-C prescribed drug treatment had higher mean total cholesterol (181.67 versus 150.65 mg/dl, *P*<0.001) and LDL-C levels (108.91 versus 81.99 mg/dl, *P*<0.001) than patients with active therapy. The frequencies of consultations with GPs and nurses were similar across both groups over the past year. All characteristics and the association measure (OR) between patients with and without active LDL-C drug prescription are shown in Table 1.

**Table 1.** Patient characteristics with active and no active LDL-C-lowering prescriptions

Characteristic^a^	Total	Without LDL-C drug prescription	With LDL-C drug prescription		
	*N* = 1936	*n* = 288 (14.88%)	*n* = 1648 (85.12%)	OR (95% CI)^b^	*P* value
Age, years, mean (SD)	74.98 (11.0)	79.91 (12.03)	74.11 (10.58)	0.95 (0.93 to 0.96)	<0.001
Age at CVD event, mean (SD)	65.26 (11.63)	68.98 (12.59)	64.61 (11.33)	0.97 (0.96 to 0.98)	<0.001
Sex, male	1363 (70.40)	140 (48.61)	1223 (74.21)	3.04 (2.35 to 3.93)	<0.001
Dependency on activities of daily living (Barthel)					
No dependence (100)	530 (27.38)	68 (23.61)	462 (28.03)	Ref	Ref
Low (60 to <100)	130 (6.71)	18 (6.25)	112 (6.80)	0.92 (0.52 to 1.60)	0.746
Moderate (40 to 55)	304 (15.70)	60 (20.83)	244 (14.81)	0.60 (0.41 to 0.88)	0.009
Severe (20 to 35)	130 (6.71)	38 (13.19)	92 (5.58)	0.36 (0.23 to 0.56)	<0.001
Total (<20)	32 (1.65)	12 (4.17)	20 (1.21)	0.25 (0.11 to 0.52)	0.001
Not registered	810 (41.84)	92 (31.94)	718 (43.57)		
Dyslipidemia blood indicators, mean (SD)					
Total cholesterol, mg/dl	155.26 (38.12)	181.67 (41.38)	150.65 (35.58)	0.98 (0.98 to 0.98)	<0.001
HDL, mg/dl	47.46 (14.96)	49.50 (13.89)	47.11 (15.11)	0.99 (0.98 to 1.00)	0.015
LDL, mg/dl	86.21 (32.12)	108.91 (34.87)	81.99 (29.64)	0.98 (0.97 to 0.98)	<0.001
Triglycerides, mg/dl	122.36 (63.29)	124.21 (57.24)	122.04 (64.30)	1.00 (1.00 to 1.00)	0.592
HbA1c, %	6.1 (0.99)	5.95 (1.07)	6.12 (0.97)	1.22 (1.03 to 1.43)	0.02
High comorbidity status (AMG ≥P85)	1535 (79.29)	206 (71.53)	1329 (80.64)	1.16 (0.77 to 1.74)	0.468
Number of chronic diseases, mean (SD)	10.42 (4.94)	10.71 (5.14)	10.37 (4.90)	0.99 (0.96 to 1.01)	0.301
Dyslipidemia	483 (24.95)	56 (19.44)	427 (25.91)	1.45 (1.06 to 1.98)	0.02
Hypertension	1213 (62.65)	190 (65.97)	1023 (62.08)	0.84 (0.65 to 1.10)	0.21
Chronic kidney disease	570 (29.44)	107 (37.15)	463 (28.09)	0.66 (0.51 to 0.86)	0.002
Type 2 diabetes mellitus	606 (31.30)	64 (22.22)	542 (32.89)	1.72 (1.28 to 2.31)	<0.001
Depression or anxiety	807 (41.68)	119 (41.32)	688 (41.75)	1.02 (0.79 to 1.31)	0.894
Chronic heart failure	275 (14.20)	49 (17.01)	226 (13.71)	0.78 [0.55to1.09]	0.145
COPD	282 (14.57)	38 (13.19)	244 (14.81)	1.14 [0.79to1.65]	0.481
Obesity	469 (24.23)	58 (20.14)	411 (24.94)	1.29 (0.95 to 1.77)	0.102
Tobacco smoke	199 (10.28)	24 (8.33)	175 (10.62)	1.31 (0.84 to 2.04)	0.238
Prescribed drugs, total, mean (SD)	6.54 (2.89)	4.49 (2.97)	6.87 (2.74)	1.39 (1.32 to 1.47)	<0.001
Consultations last year, mean (SD)					
Family physician	3.51 (3.50)	3.62 (3.53)	3.50 (3.49)	0.99 (0.95 to 1.03)	0.597
PHC nurse	4.82 (6.86)	4.30 (7.62)	4.91 (6.74)	1.02 (0.99 to 1.04)	0.196
Consultation with cardiologist last year	452 (23.35)	80 (27.78)	372 (22.57)	0.76 (0.57 to 1.01)	0.058

^a^Data given as *n* (%) unless otherwise stated. ^b^OR is derived from univariate linear or logistic regression models. AMG = adjusted morbidity group. CVD = cardiovascular disease. COPD = chronic obstructive pulmonary disease. HDL-C = high-density lipoprotein cholesterol. LDL-C = low-density lipoprotein cholesterol. OR = odds ratio. PHC = primary health care. SD = standard deviation.

According to 2016 and 2019 ESC and EAS guidelines (thresholds of<70 mg/dl and <55 mg/dl), 35.02% and 12.65% of participants, respectively, attained LDL-C target levels. Multivariate-adjusted models showed associations between LDL-C target levels with sex and type 2 diabetes mellitus. Better LDL-C attainment to the 2019 ESC and EAS guidelines thresholds was associated with type 2 diabetes mellitus (OR 0.55, 95% CI = 0.42 to 0.73) and sex (87.35% of males versus 67.95% of females). Table 2 shows the unadjusted and multivariate-adjusted OR for the association between factors and LDL-C threshold attainment. The attainment of the LDL-C threshold, based on the 2016 ESC and EAS guidelines, was defined as <70 mg/dl, while the threshold based on the 2019 ESC and EAS guidelines was set at <55 mg/dl. The results of LDL-C-lowering based on the drug prescribed intensity showed that 33.5% (*n* = 552) of participants were prescribed a low- or mild-intensity LDL-C-lowering prescription (data not shown). The multivariate-adjusted ordinal logistic regression model in Table 3 shows that males had a 1.57 OR (95% CI = 1.27 to 1.94) for receiving higher-intensity LDL-C-lowering treatments. Furthermore, age was inversely associated with the intensity of LDL-C drug therapy (OR 0.96, 95% CI = 0.95 to 0.97). Table 3 shows sex-based disparities in prescribed LDL-C-lowering treatment across drug intensity groups: females consistently had higher LDL-C levels than males across all groups.

**Table 2.** Factors associated with LDL-C threshold attainment based on 2016 and 2019 ESC and EAS guidelines

Associated factor	2016 LDL-C ESC and EAS guidelines threshold						2019 LDL-C ESC and EAS guidelines threshold					
	<70 mg/dl, *n* (%)^a^	≥70 mg/dl, *n* (%)^a^	Univariate regression^b^	Multivariate adjusted^b^			<55 mg/dl, *n* (%)^a^	≥55 mg/dl, *n* (%)^a^	Univariate regression^b^	Multivariate adjusted^b^		
	**678** (35.02)	1258 **(64.98)**	OR	OR	95% **CI**	*P* value	**245** (12.65)	1691 (87.35)	OR	OR	95% **CI**	*P* value
Age, mean (SD)	73.93 (10.37)	75.54 (11.29)	1.01^c^	1.01	1.00 to 1.02	0.098	73.70 (10.67)	75.16 (11.04)	1.01^d^	1.01	1.00 to 1.02	0.227
Sex, male	560 (82.60)	803 (63.83)	0.37^d^	0.41	0.32 to 0.52	<0.001	214 (87.35)	1149 (67.95)	0.31^d^	0.34	0.23 to 0.51	<0.001
Type 2 diabetes mellitus	271 (39.97)	335 (26.63)	0.55^d^	0.59	0.48 to 0.72	<0.001	110 (44.90)	496 (29.33)	0.51^d^	0.55	0.42 to 0.73	<0.001
Tobacco smoke	82 (12.09)	117 (9.30)	0.75^d^	0.83	0.60 to 1.13	0.237	29 (11.84)	170 (10.05)	0.83			
Hypertension	433 (63.86)	780 (62.00)	0.92				157 (64.08)	1056 (62.45)	0.93			
Chronic kidney disease	206 (30.38)	364 (28.93)	0.93				79 (32.24)	491 (29.04)	0.86			
Dyslipidemia	157 (23.16)	326 (25.91)	1.16				57 (23.27)	426 (25.19)	1.11			
Obesity (*n* = 1855)	157/649 (24.19)	312/1206 (25.87)	1.01				56/237 (23.63)	413/1618 (25.53)	1.11			

^a^Data given as *n* (%) unless otherwise stated. ^b^Unadjusted and multivariate-adjusted model ORs are reported for groups that attained the LDL-C target (<70 mg/dl and <55 mg/dl).^c^P<0.01.^d^P<0.001. EAS = European Atherosclerosis Society. ESC = European Society of Cardiology. LDL-C = low-density lipoprotein cholesterol. OR = odds ratio. SD = standard deviation.

**Table 3.** Factors associated with LDL-C based on drug prescribed intensity. The order of logistic model results are based on the drug’s prescribed intensity.

Associated factors	Univariate	Multivariate adjusted		
	OR	OR	95% **CI**	*P* value
Sex, male	1.76^a^	1.57	1.27 to 1.94	<0.001
Age, years	0.96^a^	0.96	0.95 to 0.97	<0.001
Tobacco smoke	1.58^a^	1.23	0.90 to 1.67	0.188
Chronic kidney disease	0.8	1.19	0.95 to 1.48	0.127
Type 2 diabetes mellitus	0.98			
Dyslipidemia	1.18			
Hypertension	0.94			
Obesity	0.98			

^a^ *P*<0.001. LDL-C = low-density lipoprotein cholesterol.

**Figure 1.**
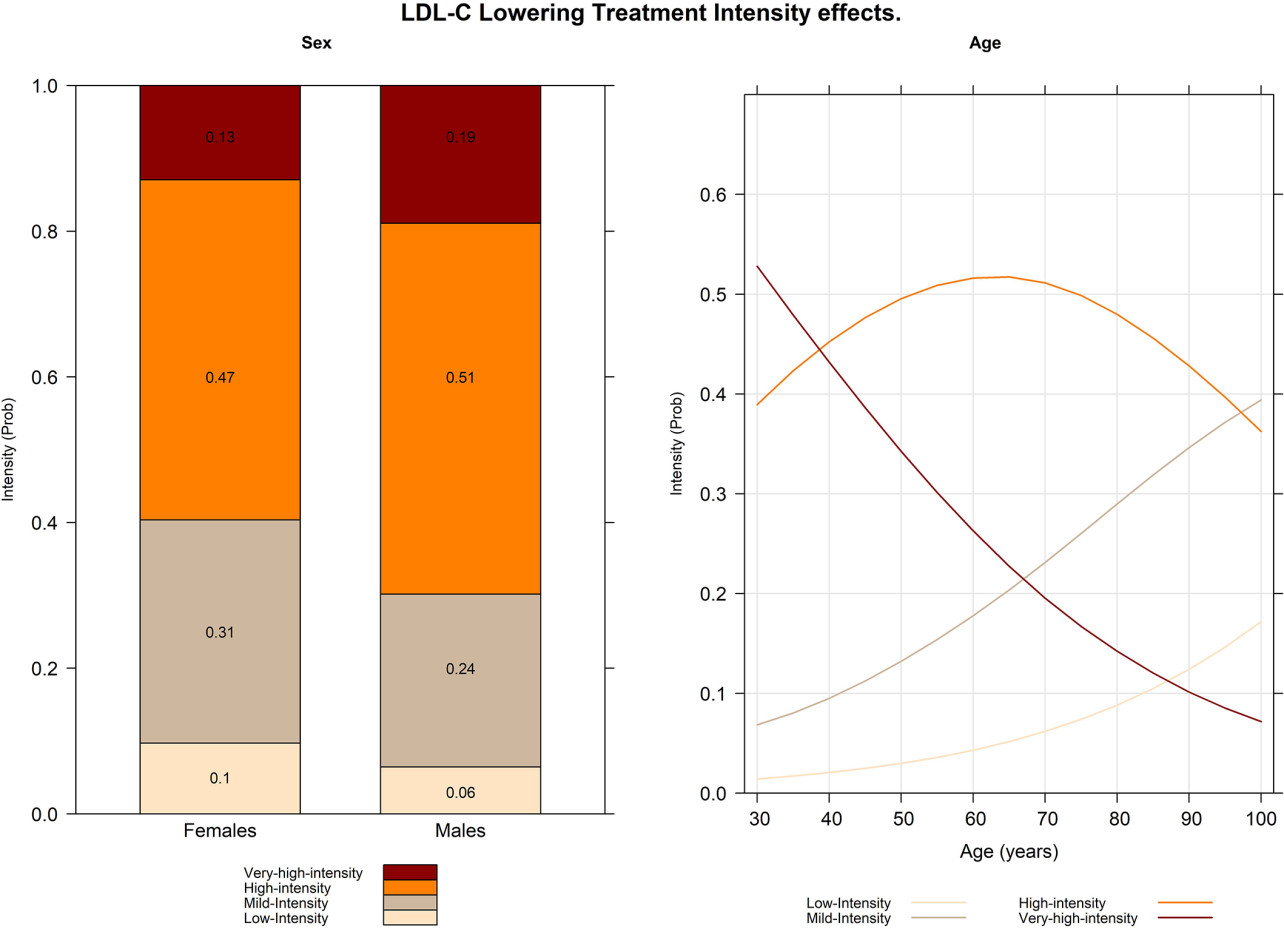
Graphical effects of the multivariate-adjusted ordered logistic model

The graphical results of the multivariate-adjusted effect model are shown in Figure 1. The stacked bar graph on the left side shows that males had a 19% and 51% likelihood of receiving very high- or high-intensity LDL-C-lowering treatment, respectively, compared with 13% and 47% for females. Conversely, females had a 10% and 31% likelihood of receiving low-intensity and mild-intensity LDL-C-lowering treatment, respectively, while for males, these probabilities were 6% and 24%. The multiline graph on the right shows age-adjusted effects. The likelihood of receiving very high-intensity LDL-C drug-lowering treatment decreased from 50.1% at age 34 years to 14.7% at age 80 years. The probability of high-intensity treatment followed a hyperbolic pattern, starting at 38.9% at age 30 years, peaking at 51.8% at age 64 years, and then declining to 47.9% at age of 80. The mild- and low-intensity treatments showed consistent incremental linear patterns, with probabilities starting at 7.5% and 1.6% at age 30 years and reaching maximums of 39.4% and 17.0%, respectively. The probability curves for the very high- and mild-intensity treatments intersected at age 69 years, with a probability of 21.3%.

## Discussion

### Summary

This study examined LDL-C-lowering drug therapy in patients necessitating secondary prevention for chronic myocardial ischaemia in an entire patient population assigned to three primary healthcare centres. Females and older patients were the majority in the group without treatment for LDL-C. Females had the least intensive treatment and higher levels of LDL-C.

### Strengths and limitations

We analysed the entire population of patients registered with a diagnosis of ischaemic heart disease. The broad coverage of the primary healthcare data source, stemming from the universal healthcare system in Spain, may contribute to a high degree of generalisability.^13,18^ However, we could not assess iPSCK9 prescriptions because we only accessed primary healthcare drug prescriptions, not hospital prescriptions managed through a different electronic system. However, an aggregated query to the central health data hub showed that only 59 patients from the participating centres (20 female and 39 male) had an active iPSCK9 or mRNA interference prescription in hospitals, representing 3.05% of all participants. Patients prescribed iPSCK9 require all LDL-C reduction therapies via hospital prescription;^19,20^ among these 59 patients, not all had a registry of ischaemic heart disease. Thus, excluding PSCK9 drug therapy in this study might have a minor impact of 3%, likely affecting no more than 12 patients (0.62%). We have focused on active drug prescriptions rather than drug adherence (that is, whether patients take medicine as prescribed). The electronic health record registry does not contain data on adherence to drug prescriptions. Therefore, examining drug adherence would require a different analytical approach beyond the scope of the current study. Another limitation is the lack of data on shared decision-making processes, which may have influenced LDL-C attainment. Finally, the study design was cross-sectional and did not capture the conditions at the time of the event but at the data extraction point, which may not have represented the patient’s initial status when the ischaemic heart disease was registered.

### Comparison with existing literature

The observed prevalence of ischaemic heart disease (2.26%) is consistent with previous reports from Spain, which have documented a gradual increase over the past decade. Specifically, the prevalence rose from 2.8% in 2009 to 3.3% in 2019.^21^ Despite frequent visits to GPs and nurses the previous year, 14.88% of patients did not receive active LDL-C-lowering treatment, predominantly female and older patients with higher mean LDL-C levels. Similar findings were observed in a study involving patients with ischaemic brain disease, where 61% were not on active treatment.^22^ A study of 19 604 patients with myocardial infarction or stroke showed that 40.8% of patients did not have LDL-C blood tests during follow-up, with women being 6.36% less likely to be tested than men.^23^ The EUROASPIRE V survey showed that 15.7% lacked active prescriptions for LDL-C-lowering therapy, with a 5.3% difference between men and women.^24^ In the field of quaternary prevention and treatment of older patients, statin therapy is recommended for coronary disease unless the patient is at the end of life or with frailty;^5^ however, the results of our study might suggest that GPs are excluding statin therapy in older patients.

The proportion of patients who attained LDL-C targeted blood levels, as per the 2016 and 2019 ESC and EAS guidelines (35.04% and 12.65%, respectively), confirms the difficulty in adhering to LDL-C recommendations. In the IMPROVE-IT clinical trial, which aimed at an LCL-C <70 mg/dl, only 37% of participants also met the LDL-C goal.^5^ Similarly, a study in seven European countries reported that only 30.9% of all patients with acute coronary syndrome had an LDL-C <70 mg/dl after 4 months of follow-up.^25^ The Da Vinci study reported that 44% of patients attained the 2016 ESC and EAS guideline’s goal (LDL-C <70 mg/dl) and 20% the 2019 ESC and EAS guideline’s goal (LDL-C <55 mg/dl); however, not all patients were visited in primary care clinics.^11^ Finally, a study in 2021 reported that only 20.1% of patients with secondary prevention achieved the 2019 ESC and EAS guidelines.^10^ While large studies confirmed poor adherence to target guidelines, our study’s attainment proportion was lower, highlighting the persistent challenge of unmet LDL-C targets and the elevated risk of major cardiovascular events.

Patients with type 2 diabetes mellitus and males had better attainment of LDL-C recommendations in the present study. The EFFECTUS survey in Italy revealed diabetes as a significant factor in attaining an LDL-C attainment as per 2016 ESC and EAS guidelines (LDL-C <70 mg/dl) with an OR of 2.73; however, no differences between males and females were reported.^26^ Another retrospective cohort study with patients who underwent a percutaneous coronary intervention in Wales (UK) showed that after a 1-year follow-up period, differences between both sexes showed that 51% of males had achieved LDL-C levels below the 2016 ESC (<1.8 mmol/l), and 27% were below the 2019 target (<1.4 mmol/l), compared with our results of 39.97% and 44.90%, respectively.^27^ A recent study in the US in individuals with established CVD and diabetes showed that statin use in males had an OR of 2.06 compared with females.^28^ Improved LDL-C attainment may be inherently linked to diabetes management, as individuals with diabetes are often subjected to more rigorous monitoring in primary care settings. This enhanced surveillance could contribute to better LDL-C control. However, it is important to note that LDL-C thresholds are not specific markers for diabetes. In the context of secondary prevention, the same LDL-C thresholds are irrespective of the previous hypercholesterolemia or diabetes. However, why patients with diabetes have a greater probability of achieving recommended LDL-C levels remains unclear and warrants further research.

Older patients and females were more likely to receive less intensive LDL-C-lowering treatment, suggesting a potential sex- and age-related bias in secondary cardiovascular disease prevention strategies. These results align with EUROASPIRE IV and V survey findings, highlighting a sex gap in lipid-lowering medication among individuals aged <70 years.^29,30^ The Tromsø study similarly demonstrated that males were likelier than females to achieve LDL-C treatment goals.^31^ Recent evidence also indicates a sex bias in LDL-C treatment intensity, with males having an adjusted OR of 0.63 (95% CI = 0.59 to 0.66) for receiving high-intensity statin prescriptions.^32^ The study’s results might corroborate the persistence of Yentl syndrome in the medical community.^33^ There is a misconception in secondary prevention that CVD is a male issue, as female’s LDL-C increases by 10%–15% after menopause, necessitating active secondary prevention.^33^ The perpetuation of sex-based healthcare inequalities by clinicians extends beyond cardiology, intersecting with how gender stereotypes influence health outcomes.^34^ The mistreatment or undertreatment intensity of females necessitating effective CVD secondary prevention is an example of a gender stereotype, which may result in more adverse health outcomes in females with cardiovascular risk.^35^

### Implications for research and practice

GPs may be withholding statin therapy from older patients despite guidelines advocating its use. This practice exacerbates the challenge of unmet LDL-C targets. Furthermore, there are persistent sex disparities in cardiovascular care, with females receiving less intensive treatment owing to ingrained stereotypes. Clinicians should prioritise guideline adherence, especially in older populations, and ensure that treatment decisions are based on objective risk factors rather than age or sex. Implementing structured risk assessments and increasing awareness of sex-based treatment disparities can help close these gaps and improve cardiovascular outcomes for all patients.

In conclusion, most patients requiring secondary prevention for ischaemic heart disease had not attained the target LDL-C, and females and older patients had insufficient drug treatment intensity and elevated LDL-C levels. These results pose a challenge for achieving LDL-C threshold recommendation targets and unveil healthcare sex and age-based inequity in secondary prevention, which may exacerbate adverse health outcomes in groups with high cardiovascular risk.

## Data

The dataset relied on in this article is available from the corresponding author on reasonable request.
